# Cognitive Predictors of Grief Trajectories in the First Months of Loss: A Latent Growth Mixture Model

**DOI:** 10.1037/ccp0000438

**Published:** 2019-09-26

**Authors:** Kirsten V. Smith, Anke Ehlers

**Affiliations:** 1 Department of Experimental Psychology, University of Oxford; Oxford Health NHS Foundation Trust, Oxford, United Kingdom; and The Loss Foundation, London, United Kingdom; 2 Department of Experimental Psychology, University of Oxford; and Oxford Health NHS Foundation Trust

**Keywords:** bereavement, latent growth mixture modeling, appraisals, memory, coping strategies

## Abstract

***Objective:*** The identification of modifiable cognitive antecedents of trajectories of grief is of clinical and theoretical interest. ***Method:*** The study gathered 3-wave data on 275 bereaved adults in the first 12–18 months postloss (T1 = 0–6 months, T2 = 6–12 months, T3 = 12–18 months). Participants completed measures of grief severity, cognitive factors (loss-related memory characteristics, negative appraisals, unhelpful coping strategies, and grief resilience), as well as measures of interpersonal individual differences (attachment and dependency). Latent growth mixture modeling was used to identify classes of grief trajectories. Predictors of class membership were identified using multinomial logistic regression and multigroup structural equation modeling. ***Results:*** Four latent classes were identified: 3 high grief classes (Stable, Low Adaptation, and High Adaptation) and a low grief class (Low Grief). When considered separately, variance in all four cognitive factors predicted membership of the high grief classes. When considered together, membership of the high grief classes was predicted by higher mean scores on memory characteristics. More negative appraisals predicted low or no adaptation from high grief severity. Losing a child also predicted membership to the stable class. Fast adaptation of high grief was predicted by a pattern of high memory characteristics but low engagement with unhelpful coping strategies. ***Conclusions:*** The findings have implications for clinical practice and point to early cognitive predictors of adaptation patterns in grief. Findings are consistent with cognitive models highlighting the importance of characteristics of memory, negative appraisals, and unhelpful coping strategies in the adaptation to highly negative life events.

## What is the public health significance of this article?

The study identifies a number of modifiable cognitive predictors that are linked to grief severity and maintenance in the first months of loss. Targeting these cognitive predictors in the first 6 months of loss may prove helpful in facilitating grief adaptation.

The death of a significant person is an inevitable part of life. The experience of mourning, set in motion after a bereavement, is thought to be an evolutionary process aimed at making the necessary alterations to relational internal working models and adapting to a life without the deceased (Bowlby, 1961). Accumulating evidence about bereavement-related difficulties has suggested that not all mourning processes resolve as intended (Jacobs, 1999; Prigerson et al., 1997, 2009), and some individuals continue to experience clinically relevant grief for many years after loss, a condition that is described in the literature as either complicated grief (CG; Prigerson, Frank, et al., 1995) prolonged grief disorder (PGD; Killikelly & Maercker, 2017a; Prigerson et al., 2009), or persistent complex bereavement disorder (PCBD; American Psychiatric Association, 2012). The wide variation in the prevalence of such prolonged severe grief reactions suggests that specific factors about the type and circumstances of the loss (Stroebe, Schut, & Stroebe, 2007), as well as relational and individual vulnerability factors have a role to play in understanding this condition (Stroebe, Folkman, Hansson, & Schut, 2006). Modifiable maintenance factors are of clinical and theoretical importance as they point to promising targets, not only in the treatment of PGD but also in the design of early intervention support strategies.

Several theoretical models (Boelen, van den Hout, & van den Bout, 2006; Maccallum & Bryant, 2013; Shear et al., 2007; Stroebe & Schut, 1999) implicate cognitive processes in the development and maintenance of PGD, in particular negative appraisals of the loss or its consequences (e.g., about the worthlessness of life without the deceased), unhelpful coping strategies (e.g., rumination), and characteristics of loss-related memories (e.g., intrusiveness, reduced specificity, and a sense of unrealness regarding the loss). In line with these models, several studies reported associations between some of these cognitive factors (appraisals, avoidance, and rumination) and PGD severity (Boelen & Lensvelt-Mulders, 2005; Boelen, van den Bout, & van den Hout, 2010; Eisma et al., 2014). Less is known about the role of characteristics of memories of the deceased and their death. Studies investigating the influence of memory on grief severity have mainly used experimental paradigms to assess general qualities of memory such as autobiographical memory specificity (Golden, 2013; Maccallum & Bryant, 2010; Robinaugh & McNally, 2013). In one exploratory study, Boelen and Huntjens (2008) reported that the frequencies of four types of specific intrusive images (i.e., positive intrusive memories of the lost person; intrusive images of the death event; reenactment fantasies; and negative images of the future) were correlated with symptoms of PGD in a sample of mourners. However, to our knowledge, no studies have yet assessed how the frequency, qualities, and consequences of loss-related memories are associated with grief severity over time.

The present study aimed to investigate to what extent a comprehensive set of potentially modifiable cognitive factors, including memory characteristics, predicts the course of grief severity in the first 12 to 18 months after loss, using the method of trajectory analysis of grief severity pioneered by Bonnano and colleagues (e.g., Bonanno, Boerner, & Wortman, 2008). We further investigated whether the modifiable cognitive factors predict grief severity over and above static moderator variables such as demographics and loss-characteristics, as well as interpersonal individual differences previously shown to be associated with grief severity such as attachment style (Maccallum & Bryant, 2018) and dependency (Bonanno et al., 2002; Mancini, Sinan, & Bonanno, 2015).

Trajectory analysis identifies subgroups or classes of individuals who show similar longitudinal patterns on a given dependent variable (e.g., grief severity profiles; Bonanno et al., 2008). This approach allows a more nuanced perspective on the way grief changes over time by finer modeling of the diversity in the experiences of grief (i.e., resilience, moderate grief with fast and slow resolution, and severe and enduring grief) than currently afforded by the diagnostic system (i.e., clinical vs. nonclinical grief; Galatzer-Levy & Bonanno, 2012) or by investigating associations with grief severity at any given point in time. A number of recent studies utilizing this approach have investigated individual covariates of class membership and found socioeconomic and loss characteristics such as low education (Lenferink, Nickerson, de Keijser, Smid, & Boelen, 2018; Nielsen, Carlsen, Neergaard, Bidstrup, & Guldin, 2018) and losing a child (Sveen, Bergh Johannesson, Cernvall, & Arnberg, 2018) to be predictive of severe and enduring grief class membership.

Trajectory analysis can help identify not only the predictors associated with grief severity, but also characteristics that distinguish between those whose grief resolves slowly or quickly. Advances in structural equation modeling (SEM) now offer the flexibility to test relational models of variables for their equivalence across trajectory groups (multigroup SEM), allowing researchers to determine whether one variable influences another uniformly by class or at differing rates (Wang & Wang, 2012). The latter method was applied in this study to test whether the relationship between appraisals, memory characteristics, and unhelpful coping strategies follows the pattern suggested by Ehlers and Clark’s (2000) cognitive model of the persistence of posttraumatic stress disorder (PTSD). This model makes specific predictions about the directionality of the maintaining cognitive factors by suggesting that excessively negative appraisals and memory characteristics induce a sense of current threat that motivates unhelpful coping strategies, which in turn prevent correction of the appraisals and the integration of new information (i.e., my loved one is not coming back) into autobiographical memory. In this study, using SEM, we modeled the first part of this hypothesis, namely that an increase in grief-related memory characteristics and appraisals would be associated with a concurrent increase in coping strategies and tested whether this relational model was equivalent across grief trajectory classes.

In summary, this study used a three-wave longitudinal design to investigate whether distinct grief trajectories exist in the first 12–18 months after loss, and if so, which factors contribute to membership of a particular trajectory. This was assessed in three ways: First we investigated whether cognitive predictors (memory characteristics, appraisals, coping strategies, and grief resilience) individually predict class membership. Next we determined in a multivariate analysis whether these associations remain significant after examining the relative contribution of background variables and interpersonal individual differences and joint variance between the cognitive predictors. Finally, a multigroup SEM was modeled to investigate whether memory characteristics and appraisals predict coping strategies in each of the grief classes.

## Method

### Participants

Participants were 275 adults (age; *M* = 46.43, *SD* = 13.24; 79% women) recruited between a few weeks and 6 months after bereavement through location targeted social media advertising and the Google content network. Eleven participants indicated they were in the first 6 months of loss at recruitment but did not complete the measures until some weeks later or reported a date of death just beyond 6 months (eight participants were 7 months and three participants were 8 months postloss). Results did not change after excluding these participants and as such they were included in the final analysis.

### Procedure

Participants were asked to complete questionnaires at recruitment (T1), and 6 (T2) and 12 (T3) months later. T1 took place on average at *M* = 2.94 months postbereavement (*SD* = 2.01, *range* = 0 to 8 months, S^2^ = 4.06), T2 on average at *M* = 9.10 months (*SD* = 2.23, *range* = 6 to 16 months, S^2^ = 4.96), and T3 on average at *M* = 14.95 months (*SD* = 2.08, *range* = 12 to 21 months, S^2^ = 4.34). All measures were completed online and collected in accordance with ethical guidelines (Smith, Thew, & Graham, 2018). The study was approved by the University of Oxford Medical Sciences Inter-Divisional Research Ethics Committee MS-IDREC-C1-2015–231.

### Measures

#### Symptom measures

Prolonged Grief Disorder Inventory (PG-13; Prigerson & Maciejewski, 2008). The PG-13 assesses the prevalence and severity of PGD symptoms (e.g., yearning for the deceased, feelings of emotional numbness/detachment from others, and feeling that a part of oneself died along with the deceased). The PG-13 is a subset of 13 items from the Inventory of Complicated Grief (ICG; Prigerson, Maciejewski, et al., 1995) and is designed to measure the PGD criteria proposed by Prigerson and colleagues (Prigerson et al., 2009). A continuous score can be derived using the sum of the score of each of the 11 grief symptoms and ranges from 11 to 55. Internal consistency of the PG-13 in this sample was excellent (α = .90).

#### Cognitive measures

The Oxford Grief (OG) study aimed to develop a comprehensive of battery of measures of cognitive and behavioral factors that contribute to the development and maintenance of PGD (loss-related memory characteristics, negative appraisals, unhelpful coping strategies, and grief resilience). Questionnaire development was informed by previous theoretical and empirical work on grief, in particular Boelen et al.’s and Eisma et al.’s work on grief appraisals and grief rumination (Boelen & Lensvelt-Mulders, 2005; Eisma et al., 2014), and PTSD, in particular appraisals and memory characteristics (Foa, Ehlers, Clark, Tolin, & Orsillo, 1999; Halligan, Michael, Clark, & Ehlers, 2003). Additional items of content not previously represented in existing measures were developed in collaboration with therapists experienced in the treatment of traumatic loss and from detailed interviews with bereaved individuals with and without PGD. The measures, which are described below, showed good to excellent psychometric properties (Smith & Ehlers, 2019).

##### Loss-related memory characteristics (OG-M)

This 27-item questionnaire is rated on a 5-point scale (0 = *not at all* to 4 = *very strongly*). Twenty-three items probed memory triggers and their consequences (e.g., I am reminded of the loss for no apparent reason.), qualities of memory (e.g., Memories of things we did together are painful.), the poor availability of positive memories (e.g., I struggle to remember positive times without [-].), and the physical impact of loss-related memories (e.g., The memories of [-]’s death make my body ache with overwhelming fatigue.). Four further items assessed qualities of unintentional memories of the loss (e.g., how distressing they were and how much they seemed to be happening now instead of in the past). The total OG-M scale demonstrated excellent internal consistency using McDonalds Omega (ω = .97).

##### Negative grief appraisals (OG-A)

This 35-item questionnaire rated on a 7-point scale (1 = *totally disagree* to 7 = *totally agree*) assesses five factors: (a) Loss of self and life (e.g., Without [-] I can never be strong again.), (b) Regret (e.g., I blame myself for things I did or did not do when [-] was alive.), (c) Catastrophic consequences of grief (e.g., If I start to cry I will not be able to stop.), (d) Loss of relationships and future (e.g., I cannot maintain previous relationships without [-].), (e). Fear of losing connection to the deceased (e.g., If I do not do everything I can to feel close to [-] I will lose them forever.). The total negative appraisals scale demonstrated excellent internal consistency (α = .97).

##### Unhelpful coping strategies (OG-CS)

This 23-item questionnaire requires participants rate on a 5-point scale (1 = *never* to 5 = *always*) how often they engage in particular strategies to cope with their loss. Items measure four factors: (a) Avoidance (e.g., I avoid places we went together.), (b) Proximity seeking (e.g. I feel compelled to surround myself with things that they liked.), (c) Grief rumination (e.g., I dwell on moments that could have changed the outcome.), and (d) Injustice rumination (e.g., I think over and over about how it could be that this happened.). The total unhelpful coping strategies scale demonstrated excellent internal consistency (α = .93).

##### Grief resilience beliefs (OG-GR)

This seven-item questionnaire rated on a 7-point scale (1 = *totally disagree* to 7 = *totally agree*) assesses statements about two content domains: (a) Continuing bonds (e.g., My memories of our time together give me confidence.), (b) Self-efficacy (e.g., Even without [-], I can deal with the ups and downs of life.). The total grief resilience scale demonstrated good internal consistency (α = .82).

#### Interpersonal individual differences measures

##### The Experiences in Close Relationships Scale Revised (ECR-S; Wei, Russell, Mallinckrodt, & Vogel, 2007)

Attachment anxiety and avoidance were measured with a validated 12-item version of the ECR-S. Participants rated their agreement with six items measuring attachment anxiety (e.g., I need a lot of reassurance that I am loved by my close loved ones.) and six items measuring attachment avoidance (e.g., I do not often worry about being abandoned) on 7-point scales (1 = *strongly disagree* to 7 = *strongly agree*). The anxious attachment subscale of the ECR-S demonstrated acceptable internal consistency (α = .77), while the avoidant attachment subscale was good (α = .83).

##### Dependency

This 16-item questionnaire asks participants to indicate on a 5-point scale (1 = *not at all true of our relationship* to 5 = *very true of our relationship*) to the extent to which the bereaved individual had depended on the deceased both emotionally (I had people other than [-] who I could confide in and share my worries with.) and practically ([-] did everything for me in our relationship.) as well as 10 items from the healthy dependency subscale of the Relationship Profile Test were included reflecting an individual’s ability to trust and turn to others in times of need (Bornstein et al., 2003). The six-item deceased dependency subscale demonstrated good internal consistency (α = .83), as did the 10-item healthy dependency subscale (α = .84).

### Data Analyses

Latent growth mixture models (LGMM) using M*plus* Version 8 (Muthén & Muthén, 2007) were used to measure heterogeneity of grief responses over time and, where possible, are reported in line with best practice (van de Schoot, Sijbrandij, Winter, Depaoli, & Vermunt, 2017). The analyses described in this paper were conducted in six steps:

1 A well-fitting single growth curve was established in the first instance to determine if a linear slope fit the overall sample trajectory (latent curve growth analysis; LCGA). Factor loadings of the time points were set to 0, 1, and 2 given the interval between measurement points was equal (6 months between time points 1 and 2, and 2 and 3).
2 One-to-five class solutions were run to determine the optimal number of classes (latent growth mixture model; LGMM; Kim, 2014; Muthén, 2003; Nylund, Asparouhov, & Muthén, 2007). We chose to adopt an LGMM approach that allows for differences in growth parameters across unobserved subpopulations or classes (Jung & Wickrama, 2008). Therefore, intercept and slope variance parameters were allowed to vary within classes.
3 The most likely class membership was then saved, merged with the original data, and used as the dependent variable in a multinomial logistic regression (MNLR) in SPSS (Version 21) to understand how classes differed on scores of the baseline individual cognitive predictors in univariate analyses. To account for these multiple comparisons, Bonferroni α adjustment set the significance level for each univariate model to *p* < .013 (α/4). The reference class was rotated to derive all possible class comparisons.
4 All four cognitive predictors measured at baseline were entered together into a MNLR controlling for background variables and interpersonal individual differences that were significantly associated with grief intensity at least one time point. A backwards elimination method was used to ensure that only background predictors that significantly improved (*p* < .05) model fit were allowed to remain in the model.
5 Multigroup SEM comprises two components: a measurement model and a structural model (Muthén, Muthén, & Asparouhov, 2017). Before determining whether classes differed on the relationships between cognitive variables at baseline it was necessary to determine whether the cognitive questionnaires (memory characteristics, negative appraisals, and coping strategies) were uniformly understood and used by each class, also known as measurement invariance (Chen, 2007; van de Schoot, Lugtig, & Hox, 2012). First, factor scores were saved for each cognitive questionnaire and imported into the data to allow for multigroup SEM.^1^ This process had the added benefit of reducing the number of parameters estimated in the SEM (Muthén & Asparouhov, 2002). For example, instead of estimating 35 items loading on five subscale factors loading on 1 second-order factor for the appraisals measure saving factor scores resulted in 1 latent factor (appraisals) and 5 observed items representing each of the subscales. This process was repeated for the coping strategies measure and the memory characteristics measure. Following this, equality constraints were applied across the classes to determine whether the assumption of measurement invariance had been confirmed. See online supplemental material for details of invariance testing.
6 Finally, an SEM was subject to a multigroup analysis in M*plus* (Version 8; Wang & Wang, 2012). Regression paths were specified from coping strategies to both memory characteristics and appraisals. This model asks whether memory characteristics and negative appraisals predict coping strategies in each grief trajectory class. The beta coefficients for the regression paths indicate the strength of these relationships for each class.

To minimize the bias associated with attrition and missing data, we used the full information maximum likelihood (FIML) approach implemented in M*plus* to estimate missing data. A majority of participants had data at all three time points (84.7%) with almost all participants answering at least two (95.6%); no participants were excluded. Covariance coverage, which measures the impact of missing data, ranged from .93 to .86 for each pair of variables, well above the minimum threshold of .10 for model convergence (Muthén, 2003). In a single growth curve analysis, the following fit indices determine adequate fit: CFI > .90, TLI > .90, RMSEA < .09, SRMR <. 08 (Hu & Bentler, 1999; Wickrama, Lee, O’Neal, & Lorenz, 2016). To determine the appropriate class solution, we examined a variety of fit statistics. In particular, the Bayesian, (BIC), sample-size adjusted Bayesian (SSBIC), and Aikaike (AIC) information criterion indices, entropy values, the Lo-Mendell-Rubin likelihood ratio test (LRT: Lo, Mendell, & Rubin, 2001), and the bootstrap likelihood ratio test (BLRT). We sought a model with lower values for the criterion indices, higher entropy values, and significant *p* values for both the LRT and the BLRT (Muthén, 2003). Fit indices in combination with theoretical interpretability guided the final model selection. Growth Mixture Models (GMM) were estimated using robust maximum likelihood method with 1,000 initial stage random starts and 120 final stage optimizations to determine if the best log-likelihood value was obtained and replicated. Finally, 100 bootstrap draws were used in the BLRT.

## Results

Zero order correlations of dichotomous and continuous background variables: gender, age, months since loss, mode of death (i.e., violent vs. nonviolent), interpersonal individual differences (anxious and avoidant attachment, dependency on the deceased and levels of healthy dependency), and cognitive predictors (memory characteristics, appraisals, coping strategies, and grief resilience) with grief intensity at recruitment (T1), 6-month follow-up (T2), and 12-month follow-up (T3) are presented in Table 1. The association of grief with the categorical background variable level of education was assessed using rank order correlations and type of loss (e.g., partner, child, parent, sibling, other relative, and close nonrelative) was assessed using repeated measures analysis of variance (ANOVA).[Table-anchor tbl1]

### Background Variables and Interpersonal Individual Differences

Gender was significantly associated with PGD symptom severity. Females had significantly higher grief intensity at all three time points. Level of education was significantly negatively associated with PGD score at each time point (T1, *r* = −.15, *p* = .02; T2, *r* = −.24, *p* < .001; T3, *r* = −.13, *p* = .04), with a lower level of education predicting a higher PGD score. When examining all levels of kinship to the deceased, the interaction between grief symptoms at T1, T2, and T3 and kinship was not significant. There was a main effect of kinship *F*(1, 5) = 12.98, *p* < .001 and post hoc tests (Hochberg’s T2) revealed significantly higher PGD scores in those who had lost children compared with those who had experienced any other loss. Those who had lost a partner reported significantly higher grief severity than those who had lost a parent, another close relative, or a close nonrelative. No other relationships significantly differed.

Attachment style and dependency were significantly associated with grief intensity at all three time points in the expected direction. Lower grief was associated with lower levels of dependency and anxious and avoidant attachment styles.

Thus, four background variables (losing a child, losing a partner, gender, and level of education) and two interpersonal individual differences (attachment style and dependency) were significantly associated with grief intensity at least one time point and, therefore, were retained in subsequent analyses.

### Cognitive Predictors

As shown in Table 1, the four cognitive predictors (memory characteristics, appraisals coping, grief resilience) measured at T1 were significantly associated with grief intensity at all three time points in the expected directions, with moderate to high effect sizes.

### Growth Curve Modeling

A single linear growth curve of PGD scores over time demonstrated an excellent fit to the data for comparative fit index (CFI) = .98, Tucker-Lewis Index (TLI) = .98, and standardized root mean square residual (SRMR) = .048, but not for root mean square error of approximation (RMSEA) = .12. The RMSEA tends not to perform well with growth curve models because of the few degrees of freedom (Kenny, Kaniskan, & McCoach, 2015). Given that three out of four fit indices demonstrated a close fit to the data it was decided to proceed to growth mixture modeling (GMM) to determine whether subsets of individuals with differing trajectories of change over time could be identified within the data.

### Latent Growth Mixture Modeling

A comparison of models with 1, 2, 3, 4, and 5 classes suggested that a 4-class model provided the best fit to the data (see Table 2) on the majority of the metrics; only entropy suggested a different class solution. The two-class solution demonstrated a nonsignificant BLRT indicating that two classes is not a better fit to the data than one class; however, BLRT returned to significance at three and four classes, suggesting improvements in model fit beyond two classes.[Table-anchor tbl2]

**Figure 1 fig1:**
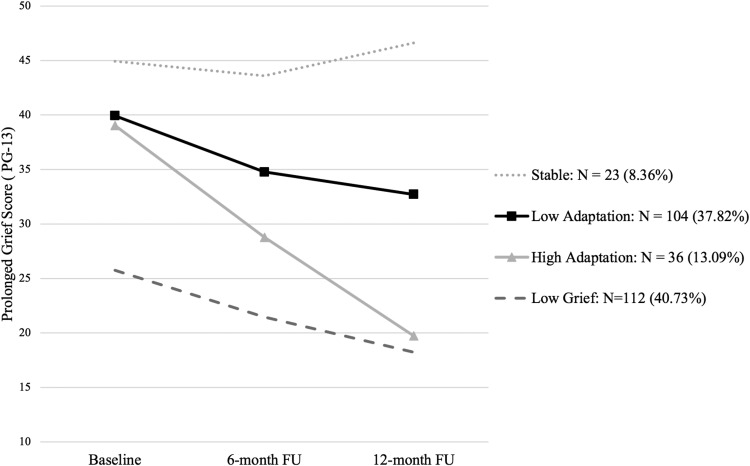
4-class Growth Mixture Model for Prolonged Grief Disorder Symptoms from baseline to 12-month follow-up (*N* = 275).

The PGD symptom trajectories for the four-class solution are illustrated in Figure 1. Each class has been given a description fitting of its trajectory. The first class ‘Stable’ has a high intercept (i.e., a high level of PGD symptoms at T1; β = 44.55, *SE* = 1.96, *p* < .001) and a slightly increasing slope (β = 1.09, *SE* = .46, *p* = .02). The next class ‘Low Adaptation’ has a high intercept (β = 39.67, *SE* = .99, *p* < .001) and a small decreasing slope (β = −3.43, *SE* = .30, *p* < .001), indicating slow resolution of grief symptoms over time. The third class, ‘High Adaptation’ class shows a high intercept (β = 38.94, *SE* = 1.58, *p* < .001), and a large decreasing slope, indicating fast resolution in their grief over time (β = −9.56, *SE* = .72, *p* < .001). The final class, ‘Low Grief’ has a low intercept (β = 25.63, *SE* = .99, *p* < .001) and a small decreasing slope, indicating modest grief symptoms that slowly decline further (β = −3.69, *SE* = .99, *p* < .001). Class demographics, loss characteristics and interpersonal individual differences in attachment style and dependency for the four grief trajectory classes are presented in Table 3.[Fig-anchor fig1][Table-anchor tbl3]

### Class Differences in Cognitive Predictors: Univariate Analyses

After α correction for multiple comparisons, variance in all four cognitive predictors (memory characteristics, appraisals, coping strategies, and grief resilience) significantly predicted class membership. Results are presented in Table 4.[Table-anchor tbl4]

Variance in all four cognitive predictor variables distinguished between the low grief class and each of the three high grief classes (stable, low adaptation, and high adaptation) in the expected directions. The largest odds ratios (*OR*s) indicating higher scores, or in the case of grief resilience lower scores, were seen in the comparison of low grief with stable, followed by those with low and high adaptation. The stable class reported significantly higher mean scores on memory characteristics, appraisals, and coping strategies, and lower scores on grief resilience, compared with both the low and high adaptation classes. Compared with the high adaptation class, the low adaptation class endorsed negative appraisals and unhelpful coping strategies more strongly, but did not differ on memory characteristics and grief resilience.

### Class Differences in Cognitive Predictors: Multivariate Analyses

MNLR investigated the unique variance explained by the cognitive predictor variables after controlling for the variance shared between them and background variables (i.e., losing a child, losing a partner, gender, and level of education) and interpersonal individual differences linked to PGD (i.e., attachment style and dependency). The backward selection method suggested that of the background and interpersonal variables, only losing a child (*p* = .02) and healthy dependency (*p* = .03) significantly contributed to the model and were, therefore, retained in the final model.

Table 5 presents the parameter estimates for the multivariate class comparisons. Memory characteristics contributed unique variance to the distinction between the low grief and all three high grief groups. Negative appraisals uniquely distinguished between the stable and low adaptation classes compared with the low grief class. The stable class was also uniquely predicted by losing a child compared with all three other groups. Compared with the high adaptation class, the low adaptation class showed greater endorsement of negative appraisals, and lower endorsement of memory characteristics, grief resilience, and healthy dependency, while the stable class reported significantly greater endorsement of negative appraisals. No other comparisons reached significance.[Table-anchor tbl5]

### Does the Relationship of Memory Characteristics and Appraisals With Coping Differ by Grief Class?

**Figure 2 fig2:**
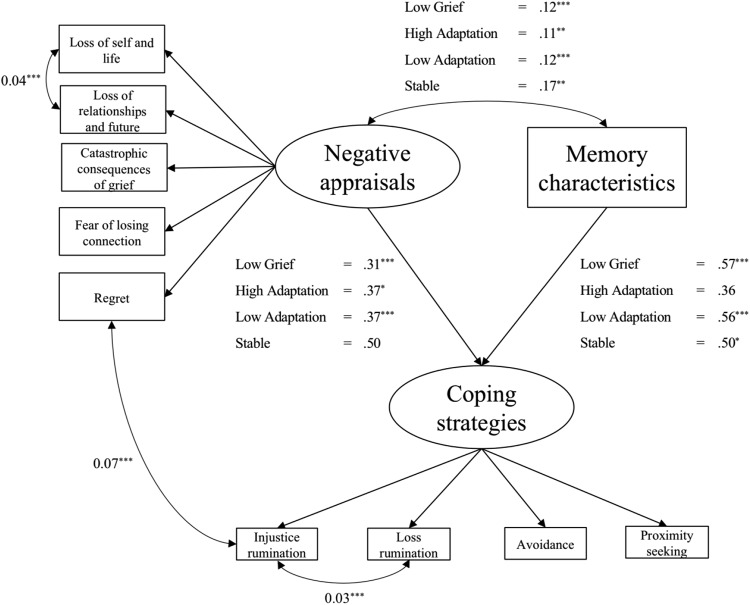
Multigroup structural equation model of relationship between cognitive predictors memory characteristics and negative appraisals on maladaptive coping. Unstandardised coefficients are reported. Correlated residuals are represented with double-headed arrows and were constrained to be equal across groups. Ovals represent latent constructs and rectangles represent raw observed scores for each subscale. * *p* < .05. ** *p* < .01. *** *p* < .001.

To test this hypothesis, the model had to first meet the assumptions of measurement invariance; this was the case for both the appraisals and coping strategies measures.^2^ The memory characteristics measure did not have a latent factor structure and as such was not subject to invariance testing. A multigroup SEM tested the hypothesis derived from Ehlers and Clark’s (2000) cognitive model for PTSD that the maintenance of high symptoms is predicted by high levels of appraisals and memory characteristics leading to unhelpful coping strategies that in turn prevent change (see Figure 2).[Fig-anchor fig2]

#### Multigroup SEM

The initial model without correlated residuals was a borderline fit to the data for CFI = .90, TLI = .90, χ^2^ = 457.54, *df* = 174 (χ^2^: *df* = 2.63); however, the RMSEA = .15 (confidence interval [CI: .14, .17]), and SRMR = .10 showed poor fit. Three correlated errors were suggested by modification indices. The first between regret beliefs and loss rumination, the second between loss of self-concept and life, and loss of relationships and future and the last between the two rumination subscales of the coping strategies scale. The two within scale correlated errors (i.e., loss of self-concept and life and loss of relationships and future; loss rumination and injustice rumination) indicate that these subscales contain items measuring similar constructs. The correlated error between regret and loss rumination might suggest that those with higher regret beliefs were more likely to engage in ruminative thinking regarding their loss in particular over and above that which is explained by the relationship between the general factors of appraisals and coping strategies. Adding these correlated residuals, which were fixed to be equal across groups, resulted in a significant improvement to model fit. The majority of fit indices suggested an excellent fit CFI = .95, SRMR = .07, χ^2^ = 324.76 *df* = 171, (χ^2^: *df* = 1.90), or near excellent TLI = .94. However, the RMSEA was above the recommended cut-off (RMSEA = .12). Overall fit was deemed suitable for interpretation. Multigroup regression coefficients are shown in Figure 2. Negative appraisals significantly correlated with memory characteristics in all four classes (Low Grief, β = .12, *SE* = .02, *p* < .001; High Adaptation, β = .11, *SE* = .03, *p* = .001; Low Adaptation, β = .12, *SE* = .02, *p* < .001; Stable, β = .17, *SE* = .06, *p* = .003).

#### Negative appraisals predict coping strategies

Negative appraisals significantly predicted unhelpful coping strategies within three of the classes (Low Grief, β = .31, *SE* = .08, *p* < .001; High Adaptation, β = .37, *SE* = .16, *p* = .02; Low Adaptation, β = .37, *SE* = .10, *p* < .001), but the relationship did not reach significance in the Stable class (β = .50, *SE* = .27, *p* = .06). Given the larger β coefficient of the regression between appraisals and coping strategies of the stable group (β = .50) compared with the other classes the lack of significance is likely because of a larger *SE* in the Stable class because of small sample size.

#### Memory characteristics predict coping strategies

Loss-related memory characteristics significantly predicted unhelpful coping strategies within three of the classes (Low Grief, β = .57, *SE* = .08, *p* < .001; Low Adaptation, β = .56, *SE* = .10, *p* < .001; Stable, β = .50, *SE* = .25, *p* = .049). The high adaptation class did not show a significant predictive relationship (β = .36, *SE* = .20, *p* = .07) suggesting that, for individuals whose grief starts high but resolves quickly, a rise in loss-related memory characteristics did not predict a rise in unhelpful coping strategies.

## Discussion

This study investigated the role of cognitive factors in predicting trajectories of grief intensity in the first months of loss in a sample of 275 bereaved individuals. Four distinct grief trajectories were identified: Low Grief (*N* = 112), High Adaptation (*N* = 36), Low Adaptation (*N* = 104), and Stable (*N* = 23). The number of participants per trajectory indicated that the vast majority of bereaved individuals saw resolution to their grief over the course of the study. The stable class whose grief severity remained high by the end of the study only comprised 8.4% of the total sample. This is in line with previous research that suggests rates of PGD are between 5 and 10% in the general population (Prigerson et al., 2009). Similarly, 40.7% of the sample demonstrated low levels of grief throughout the course of the study, which is broadly in line with findings that suggested resilience to grief is shown by 45–65% of grievers (Bonanno et al., 2002; Mancini, Bonanno, & Clark, 2011).

Measures of cognitive factors taken at the first assessment (i.e., loss-related memory characteristics, negative grief-related appraisals, coping strategies, and grief resilience) derived from theories of PGD were tested for their ability to predict grief intensity over time. In univariate analyses, variance in all four cognitive predictors distinguished between the high grief classes and the low grief class, indicating that these cognitive factors may be useful in identifying those likely to suffer prolonged clinical and nonclinical grief reactions. The highest endorsement of cognitive predictors, apart from grief resilience, which was the lowest, was observed in the stable class, followed by the low adaptation, and then the high adaptation class. This suggests that the increases in cognitive predictors are closely related to the intensity and chronicity of grief reactions. An interesting pattern of cognitive differences were observed when investigating the cognitive correlates of the class that showed high initial grief but fast resolution. In the univariate analysis, the high adaptation class had lower scores on negative appraisals and unhelpful coping strategies compared with the low adaptation class, whereas memory characteristics and grief resilience did not differ. This suggests that while the presence of loss-related memory characteristics and low resilience beliefs predict clinically significant grief symptoms in the first 6 months of loss, it is the severity of negative appraisals and unhelpful coping strategies that predict slow adaptation. The multivariate analysis supported this pattern of findings in that higher negative appraisals contributed uniquely to the distinction between the high adaptation class and the two classes with no or small changes in grief intensity over time (low adaptation, stable) when controlling for the variance shared between the predictors and after examining the relative contribution of background variables and interpersonal individual differences of importance.

Thus, across analyses lower scores on negative appraisals were predictive of greater adaptation to loss, that is, either low grief or fast resolution of high grief. Higher appraisals increased the odds of being in the low adaptation class or the stable class compared with the high adaptation and low grief classes. Together, these findings indicate that low negative appraisals in people with high grief symptoms are a good prognostic sign, whereas highly negative appraisals are linked to the maintenance of grief symptoms. These results support previous findings that negative appraisals about the self, the world and others after loss are linked with concurrent and prospective grief severity (Boelen, van den Bout, & van den Hout, 2006; Bonanno et al., 2002).

Loss-related memory characteristics were related to grief severity and were significantly elevated in all three high grief classes in comparison to the low grief class in the univariate analyses. High initial memory characteristics did not necessarily mean long-term prolonged grief, as indicated by the pattern of differences between the high and low adaptation classes (no difference in univariate analysis and greater memory characteristics in the high adaptation class in the multivariate analysis). It is possible that the high memory characteristics in the high adaptation group reflect active engagement with loss-related memories, a process that that cognitive theory would suggest is helpful in consolidating and contextualizing traumatic memories into the autographical (Boelen, van den Hout, et al., 2006; Ehlers & Clark, 2000) or attachment-related memory base (Shear et al., 2007). To further explore this possibility a multigroup SEM, based on the cognitive model for PTSD, tested whether appraisals and memory characteristics differed in their predictive utility on coping strategies by grief class. Results showed that while the stable class showed the strongest predictive association between appraisals and coping strategies, the other three classes showed a relationship of similar strength. In contrast, all but the high adaptation class showed a significantly predictive relationship between memory characteristics and coping strategies. This result provides some explanation for the comparable starting severity of the high and low adaptation classes and their disparate trajectories. Perhaps the magnitude of loss-related memory characteristics explains the high starting grief severity observed in the high adaptation class, as this was the only cognitive predictor significantly elevated in this class compared with the low adaptation class. However, there was no predictive relationship between memory characteristics and unhelpful coping strategies in the high adaptation class, meaning that initial levels of grief-related memory characteristics in the bereavement process did not result in unhelpful coping. This may explain why these individuals were able to adapt to their grief over time.

Previous research has found grief-related appraisals and coping strategies to be important in the prediction of PGD (Boelen, de Keijser, & Smid, 2015; Boelen & Eisma, 2015; Boelen, van den Bout, & van den Hout, 2003a) but few studies have investigated a number of cognitive predictors for their relative importance (Boelen, van den Bout, et al., 2006; Boelen et al., 2010; Boelen & van den Hout, 2008), and none have investigated loss-related memory characteristics. Our results are in line with those of Boelen and colleagues, in that appraisals, and to a certain extent coping strategies, distinguished between the low and high grief groups. However, our results also offer support for the hypothesis that loss-related memory characteristics have a unique role to play in predicting grief severity over time. We further demonstrated the utility of cognitive–behavioral factors over and above not only background variables and some loss characteristics (in line with Boelen et al., 2006), but also over and above interpersonal individual differences such as dependency and attachment style (Denckla, Bornstein, Mancini, & Bonanno, 2015; Mancini, Robinaugh, Shear, & Bonanno, 2009).

Of the characteristics of the loss, losing a child was the only variable that contributed to model fit in the multivariate analysis and, therefore, remained in the model. Other variables such as mode of death, and age, which have previously been found to influence the course of grief (Stroebe & Schut, 2001), were not predictive of grief severity in the present sample. Child loss was the strongest unique predictor, along with memory characteristics and appraisals, of membership to the severe and enduring grief class (Stable), 35% of this group had lost a child. Our results are supported by a recent trajectory analysis that found losing a child to be the strongest predictor of chronic grief in a sample of bereaved Swedish survivors of the 2004 Indian Ocean tsunamis (Sveen et al., 2018). One explanation for this might be that parent–child bonding is generally accepted as the strongest of the attachment relationships (Sanders, 1980), another might be that losing a child violates a number of closely held assumptions regarding caregiver responsibility and the expected course of life (Zetumer et al., 2015). These results implicate bereaved parents as a particularly vulnerable group, a large proportion of whom see no resolution to their grief in the first 12–18 months after loss.

Of the interpersonal individual differences investigated, only healthy dependency contributed significant variance to group membership, with the high adaptation class reporting healthier dependency style compared with the low adaptation class. These results fit with previous research that found that preloss interpersonal dependency and dependency on the deceased was associated with a chronic grief and chronic depression trajectory (Bonanno et al., 2002). Our results suggest that alongside negative appraisals and low grief resilience beliefs, low interpersonal dependency can contribute to slow resolution in those with high grief.

The current study extends the literature on cognitive–behavioral factors and their role in predicting grief severity over time. Nonetheless, there are some limitations that are worth noting. First, while overall the sample size was sufficient for LGMM analyses the size of each class may have limited the power to detect group differences on some variables in the multivariate analysis (Kline, 2015; Wang & Wang, 2012). Examination of beta coefficients and standard errors suggested that sample size played a role in the failure to find a predictive relationship between appraisals and coping strategies in the stable group in the multigroup SEM. Second, the sample was predominantly White and female and future research should aim to replicate these results in a more diverse sample to ensure racial and gender equality. Third, this study was undertaken before the adoption of the International Classification of Diseases-11th Revision (ICD-11) criteria for PGD (Killikelly & Maercker, 2017a) and utilizes the PG-13 that is based on the PGD-2009 criteria (Prigerson et al., 2009). While previous research suggested that the conceptualisations of ICD-11 PGD, PGD-2009, and PCBD are diagnostically identical (Maciejewski, Maercker, Boelen, & Prigerson, 2016), a recent study has suggested that PGD-2009 underdiagnoses participants with severe and impairing grief compared with the ICD-11 criteria (Mauro et al., 2018). However, given that the PGD-2009 criteria have been cited as the empirical basis for, and most closely representing, the ICD-11 criteria for PGD (Killikelly & Maercker, 2017a, 2017b) we hope our results can prove useful in identifying relevant cognitive predictors for severe and enduring grief, especially as we used it as a continuous measure to determine trajectories rather than for diagnostic classification. Future studies using validated measures of the ICD-11 PGD criteria or the PCBD criteria would be helpful in determining the generalizability of these results. Fourth, the use of self-report questionnaires to measure both dependent and independent variables may have contributed to shared method variance. Future research should seek to use clinical interviews of grief severity that include assessment of social, domestic, or occupational functioning that would allow for stronger conclusions regarding the impact of cognitive risk factors on psychopathology. Fifth, the questionnaires used in this study aimed to provide a comprehensive battery of cognitive predictors relevant to the experience of PGD. The measures were sensitive in showing group differences in the present study and demonstrated good to excellent psychometric validity (Smith & Ehlers, 2019). However, a limitation is that no comparison to existing measures of appraisals (GCQ: Boelen & Lensvelt-Mulders, 2005; PTCI; Foa et al., 1999) and rumination (UGRS; Eisma et al., 2014) after bereavement is available yet, so that it remains unclear whether the group differences would replicate with these established measures. However, some of the content of the measures and their factors overlap with the existing measures (e.g., negative beliefs about the self, life, and grief as well as injustice rumination) and findings converge with previous findings (Boelen, van den Bout, et al., 2006; Eisma et al., 2015) suggesting their validity. Sixth, the cross-sectional nature of the data in the multigroup SEM limits conclusions regarding causation. Future research should seek to confirm these findings using longitudinal data such as ecological momentary assessment (EMI) or via experimental studies in which the direction of causality is controlled.

Notwithstanding these limitations, this study provided evidence of divergent grief trajectories in the first 12 to 18 months after bereavement. Cognitive predictors were able to distinguish between those in low and high grief groups, and those with fast and slow or no resolution of their grief. Further, by showing that our cognitive measures predict grief trajectories over and above sociodemographic variables, loss-characteristics, and interpersonal individual differences, we can be confident that a focus on cognitive factors is likely to prove helpful in explaining grief adaptation. They offer targets for psychological treatment and suggest that modifying the individual’s negative appraisals, unhelpful coping strategies, and characteristics of their grief-related memories is likely to be helpful in addressing disabling persistent grief reactions. Finally, while the vast majority of individuals experienced reductions in their grief over time, this study points to cognitive factors that, if targeted early, in first 6 months of loss, may prove useful in facilitating grief adaptation.

## Supplementary Material

10.1037/ccp0000438.supp

## Figures and Tables

**Table 1 tbl1:** Zero Order Correlations of PGD at Baseline (T1), 6-Month Follow-Up (T2), and 12-Month Follow-Up, (T3), Background Variables, Interpersonal Individual Differences, and Cognitive Measures

Variable	1	2	3	4	5	6	7	8	9	10	11	12	13	14	15	16
1. T1-PG-13	—															
2. T2-PG-13	.82***	—														
3. T3-PG-13	.76***	.85***	—													
4. Sex	.21***	.22***	.19**	—												
5. Age	−.02	.03	.10	.00	—											
6. Months	.04	.07	.12	−.06	.08	—										
7. Partner	.21**	.18**	.19**	−.08	.28***	.02	—									
8. Child	.25***	.30***	.33***	.07	.23***	.13*	−.20**	—								
9. Mode	.11	.03	.09	.11	.01	.03	.05	.20**	—							
10. AxECR-S	.30***	.29***	.20**	.00	−.16**	.02	−.10	.02	−.11	—						
11. AvECR-S	.31***	.28***	.23***	.06	−.14*	.07	.04	−.03	−.01	.32***	—					
12. InD	−.42***	−.40***	−.38***	.14*	−.16*	−.05	−.45***	.12	.05	−.09	−.15*	—				
13. HD-RPT	−.41***	−.36***	−.38***	−.11	.14*	−.02	.02	−.08	.00	−.50***	−.62***	.19**	—			
14. OG-M	.86***	.76***	.70***	.20**	−.03	−.02	.22***	.20**	.11	.24**	.25***	−.43***	−.31***	—		
15. OG-A	.81***	.74***	.73***	.09	−.03	.11	.25***	.24***	.06	.42***	.38***	−.51***	−.49***	.81***	—	
16. OG-CS	.79***	.71***	.70***	.13*	−.09	.07	.15*	.26***	.10	.31***	.26***	−.41***	−.32***	.83***	.80***	—
17. OG-GR	−.50***	−.44***	−.44***	−.15*	−.05	−.05	−.15*	−.15*	−.14*	−.26***	−.38***	.27***	.54***	−.47***	−.61***	−.41***
*Note*. Zero order correlations for longitudinal data (*n* = 275). PG-13 = Prolonged Grief Disorder scale; months = months since loss; partner = partner loss; child = child loss; mode = mode of death; AxECR-S = Anxious Attachment Style Experiences in Close Relationships–Short version; AvECR-S = Avoidant Attachment Style Experiences in Close Relationships–Short version; InD = independence from deceased; HD-RPT = healthy dependency subscale–The Relationship Profile Test; OG-M = loss-related memory characteristics; OG-A = negative grief-related appraisals; OG-CS = maladaptive coping strategies; OG-GR = grief resilience. Pearson’s correlation (two-tailed).																
* *p* < .05. ** *p* < .01. *** *p* < .001.																

**Table 2 tbl2:** Fit Indices for Latent Class Growth Analysis Examining Prolonged Grief Disorder Symptoms From Baseline to 12 Month Follow-Up (N = 275)

Number of classes	AIC	BIC	SSBIC	Entropy	VLMR-LRT *p* value	BLRT *p* value	Sample size by class based on most likely membership
1-Class	5188	5217	5191	—	—	—	275
2-Class	5167	5207	5172	.67	<.001	.14	81/194
3-Class	5128	5175	5133	.82	<.001	<.001	21/104/150
4-Class	5109	5167	5117	.77	<.001	.01	112/36/104/23
5-Class	5109	5177	5117	.74	.30	.79	99/55/67/32/22
*Note*. AIC = Akaike information criterion; BIC = Bayesian information criterion; VLMR = Vuong-Lo-Mendell-Rubin test; BLRT = bootstrap likelihood ratio test.							

**Table 3 tbl3:** Demographics, Loss Characteristics, Interpersonal Individual Characteristics, and Cognitive Factors by Grief Trajectory Class

	Low grief	High adaptation	Low adaptation	Stable
Variable	(*n* = 112)	(*n* = 36)	(*n* = 104)	(*n* = 23)
Demographics				
Age in years *M* (*SD*)	45.18 (13.91)	44.53 (11.88)	47.37 (12.99)	51.30 (12.18)
Gender *N* (%) female	81 (72.3)	30 (83.3)	85 (81.7)	20 (87.0)
Highest level of education *N* (%)				
No qualification	2 (1.8)	1 (2.8)	3 (2.9)	3 (13.0)
High school education	34 (30.4)	9 (25.0)	37 (35.6)	5 (21.7)
University degree	43 (38.4)	19 (52.8)	41 (39.4)	8 (34.8)
Postgraduate degree	33 (29.5)	7 (19.4)	23 (22.1)	7 (30.4)
Ethnicity *N* (%) White	103 (92.0)	35 (97.2)	94 (90.4)	22 (95.7)
Loss characteristics				
Months since loss at baseline *M* (*SD*)	2.90 (2.07)	2.53 (1.79)	2.98 (2.01)	3.61 (2.02)
Who died*? N* (%)				
Spouse/partner	21 (18.8)	14 (38.9)	39 (37.5)	9 (39.1)
Child	2 (1.8)	1 (2.8)	13 (12.5)	8 (34.8)
Sibling	9 (56.3)	2 (5.6)	4 (3.8)	1 (4.3)
Parent	51 (45.5)	15 (41.7)	36 (34.6)	3 (13.0)
Other relative	25 (22.3)	3 (8.3)	9 (8.7)	2 (8.7)
Close nonrelative	4 (3.6)	1 (2.8)	3 (2.9)	0 (.0)
Length of relationship (months) *M* (*SD*)	431.97 (182.15)	348.33 (197.67)	382.64 (179.29)	338.91 (208.80)
How did they die? *N* (%)				
Nonviolent	104 (92.9)	29 (80.6)	97 (93.3)	18 (78.3)
Violent (e.g. accident, homicide, suicide, drug overdose, and medical negligence)	8 (7.1)	7 (19.4)	7 (6.7)	5 (21.7)
Interpersonal individual differences				
Anxious attachment *M* (*SD*)	19.13 (6.96)	20.66 (7.63)	22.30 (7.60)	24.13 (10.14)
Avoidant attachment *M* (*SD*)	16.50 (7.32)	17.17 (8.05)	20.46 (7.54)	19.83 (6.47)
Independence from deceased *M* (*SD*)	25.03 (4.30)	21.17 (6.12)	20.43 (6.17)	18.65 (6.15)
Healthy dependency *M* (*SD*)	35.33 (6.99)	35.25 (6.24)	30.97 (7.27)	28.22 (7.09)
Cognitive predictors				
Memory characteristics *M* (*SD*)	33.47 (17.84)	66.12 (16.44)	66.60 (18.71)	78.56 (21.40)
Appraisals *M* (*SD*)	78.80 (27.80)	115.17 (39.19)	136.49 (38.94)	167.65 (42.91)
Coping strategies *M* (*SD*)	40.83 (10.43)	57.33 (15.44)	60.00 (16.97)	75.08 (19.08)
Grief resilience *M* (*SD*)	37.60 (6.79)	33.00 (8.86)	32.66 (8.67)	26.39 (8.32)
*Note*. Violent loss is defined as bereavement resulting from human (in)action (Currier, Irish, Neimeyer, & Foster, 2015).				

**Table 4 tbl4:** Univariate Analyses of Cognitive Predictors on Class Membership

	Reference group					
	Low grief (LG)		High adaptation (HA)		Low adaptation (LA)	
Comparison group	*B* (*SE*)	*OR* [95% CI]	*B* (*SE*)	*OR* [95% CI]	*B* (*SE*)	*OR* [95% CI]
HA						
Memory characteristics	.09 (.01)	1.09 [1.06, 1.12]***				
Appraisals	.03 (.01)	1.03 [1.02, 1.05]***				
Coping strategies	.10 (.02)	1.11 [1.07, 1.14]***				
Grief resilience	−.08 (.02)	.93 [.88, .97]**				
LA						
Memory characteristics	.09 (.01)	1.10 [1.07, 1.13]***	.01 (.01)	1.01 [.98, 1.03]		
Appraisals	.05 (.01)	1.05 [1.04, 1.06]***	.02 (.01)	1.02 [1.01, 1.03]**		
Coping strategies	.13 (.02)	1.13 [1.10, 1.17]***	.02 (.01)	1.02 [1.00, 1.05]*		
Grief resilience	−.09 (.02)	.92 [.89, .95]***	−.01 (.02)	.99 [.95, 1.04]		
S						
Memory characteristics	.13 (.02)	1.13 [1.09, 1.18]***	.04 (.02)	1.04 [1.01, 1.07]*	.03 (.01)	1.03 [1.01, 1.06]*
Appraisals	.06 (.01)	1.07 [1.05, 1.08]***	.03 (.01)	1.03 [1.02, 1.05]***	.02 (.01)	1.02 [1.00, 1.03]**
Coping strategies	.16 (.02)	1.17 [1.13, 1.22]***	.06 (.02)	1.06 [1.03, 1.09]***	.03 (.01)	1.03 [1.01, 1.06]*
Grief resilience	−.16 (.03)	.86 [.81, .91]***	−.08 (.03)	.92 [.87, .98]*	−.07 (.03)	.93 [.89, .98]**
*Note*. CI = confidence interval; *OR* = odds ratio; S = Stable.						
* *p* ≤ .05. ** *p* ≤ .01. *** *p* ≤ .001.						

**Table 5 tbl5:** Multivariate Analysis Parameter Estimates of Class Comparisons for Cognitive Predictor Variables, Child Loss, and Healthy Dependency

	Reference group					
	Low grief (LG)		High adaptation (HA)		Low adaptation (LA)	
Comparison group	*B* (*SE*)	*OR* [95% CI]	*B* (*SE*)	*OR* [95% CI]	*B* (*SE*)	*OR* [95% CI]
HA						
Memory characteristics	.09 (.02)	1.09 [1.05, 1.14]***				
Appraisals	.00 (.01)	1.00 [.98, 1.02]				
Coping strategies	.02 (.03)	1.02 [.97, 1.07]				
Grief resilience	.04 (.04)	1.05 [.98, 1.12]				
Child loss = 0	.07 (1.37)	1.07 [.07, 16.33]				
Healthy dependency	.06 (.04)	1.06 [.98, 1.14]				
LA						
Memory characteristics	.05 (.02)	1.05 [1.02, 1.08]**	−.04 (.02)	.96 [.93, .99]*		
Appraisals	.03 (.01)	1.03 [1.01, 1.04]**	.03 (.01)	1.03 [1.01, 1.04]**		
Coping strategies	.04 (.02)	1.04 [1.00, 1.08]^†^	.02 (.02)	1.02 [.98, 1.07]		
Grief resilience	−.05 (.03)	.95 [.89, 1.02]	−.10 (.03)	.91 [.85, .97]**		
Child loss = 0	−1.38 (.96)	.25 [.04, 1.64]	−1.45 (1.10)	.24 [.03, 2.04]		
Healthy dependency	−.05 (.03)	.96 [.90, 1.02]	−.10 (.04)	.90 [.84, .97]**		
S						
Memory characteristics	.05 (.03)	1.06 [1.00, 1.11]*	−.04 (.03)	.97 [.92, 1.02]	.01 (.02)	1.01 [.96, 1.05]
Appraisals	.03 (.01)	1.03 [1.01, 1.06]*	.03 (.01)	1.03 [1.00, 1.06]*	.01 (.01)	1.01 [.98, 1.03]
Coping strategies	.05 (.03)	1.05 [.99, 1.11]	.03 (.03)	1.03 [.98, 1.09]	.01 (.02)	1.01 [.96, 1.06]
Grief resilience	.00 (.05)	1.00 [.91, 1.11]	−.04 (.05)	.96 [.87, 1.06]	.05 (.04)	1.06 [.97, 1.15]
Child loss = 0	−2.51 (1.07)	.08 [.01, 66]*	−2.58 (1.16)	.08 [.01, .73]*	−1.13 (.57)	.32 [.11, .99]*
Healthy dependency	−.05 (.05)	.95 [.86, 1.05]	−.10 (.05)	.90 [.82, 1.00]	−.00 (.04)	1.00 [.92, 1.08]
*Note*. CI = confidence interval; *OR* = odds ratio; S = Stable.						
^†^ *p* < .10. * *p* < .05. ** *p* < .01. *** *p* < .001.						

## Footnotes

1 Multi-group SEM in M*plus* requires that all groups have the same range of observations. This is not expected in community samples investigating mental health problems. For example, a nonclinical group is unlikely to endorse higher items on the scale and vice versa.

2 Detailed steps of invariance testing can be found in online supplemental material.
