# Supplementary material for: Cognitive Predictors of Grief Trajectories in the First Months of Loss: A Latent Growth Mixture Model
Source: J Consult Clin Psychol. 2019 Sep 26;88(2):93–105. doi: 10.1037/ccp0000438 (PMC6939605; doi:10.1037/ccp0000438)
Supplement: Supplementary file 2 [file CCP-2018-1485Supplimentary_FINAL.docx]

**Confirming the measurement model for the multi-group structural equation model.**

##

## Statistical Analyses

Multi-group structural equation modeling (SEM) is made up of two components: a measurement model and a structural model (Muthén, Muthén, & Asparouhov, 2017). Crucially the measurement model allows the researcher to confirm that the measurement instrument, in this case cognitive questionnaires, are being used in a uniform fashion by the groups in question. Before comparing groups on their scores the researcher must determine whether the scores mean the same thing for each group, also known as measurement invariance (Van de Schoot, Lugtig, & Hox, 2012). Following this the structural model allows the researcher to compare groups of individuals on their scores on predictors and the relational paths between predictors. The following fit indices determine adequate fit: CFI>.90, TLI>.90, RMSEA<.09, SRMR<.08, and a chi square to degrees of freedom ratio of less than 3:1 (Hu & Bentler, 1999; Wickrama, Lee, O’Neal, & Lorenz, 2016).

### Measurement model.

Measurement invariance. When a series of equality constraints (i.e. configural invariance, metric invariance, and scalar invariance) are placed on the model in succession and the model fit does not decrease substantially then assumptions of measurement invariance are said to have been met (Chen, 2007; Van de Schoot et al., 2012). Configural invariance confirms that the components that comprise the factor are the same for all groups (i.e. avoidance, proximity seeking, loss rumination, and injustice rumination make up the latent factor coping behaviours). Metric invariance assumes that the strength of the relationship or the ‘weighting’ of each component on the factor is the same across groups confirming that respondents across groups attribute the same meaning to the latent factor (Van de Schoot et al., 2012). Finally, when scalar invariance is confirmed the intercepts of each item can be fixed across groups without compromising model fit. These assumptions taken together allow the researcher to compare groups on their scores on a particular instrument by defining and testing the structural model.

**Results**

## Measurement model

Multi-group analysis of categorical indicators in Mplus requires that all items on a scale be observed in all groups (Muthén & Muthén, 2007). Community data investigating mental health problems rarely fits these criteria, as those suffering from a clinical problem are unlikely to endorse the lower items on the scale and vice versa. Clinical groups will tend to cluster around the higher range while the non-clinical groups cluster around lower items. Therefore, in order to meet the requirements for multi-group SEM, a two step process was employed to model first-order confirmatory factor analyses (CFA) for the negative appraisals, unhelpful coping strategies scales and a single item indicator for the loss-related memory characteristics scale. First, second order factors were modeled separately for both appraisals (i.e. 35 items loading on 5 subscale factors loading on 1 second order factor) and coping strategies (i.e. 23 items loading on 4 factors loading on 1 second order factor), and a first order factor was modeled for memory characteristics (i.e. 27 items loading on 1 first order factor). The factor structure of these scales was confirmed in forthcoming studies (Smith & Ehlers, 2019). Second, latent variable factor scores for the above models were saved as new variables and reimported into the dataset to be used in subsequent analyses. This process turns a second factor model into a first factor, or in the case of memory characteristics, a one factor latent model into a single item measure, by subsuming the item responses into a factor score. This process substantially reduced the amount of parameter estimates making it more suitable for the study’s sample size.

The first order CFA scalar model for the appraisals scale was an acceptable fit to the data on most indices (χ^2^ = 68.84 df = 24, χ^2^:df = 2.04 , CFI = .97; TLI = .94; SRMR=.04) but above the threshold for acceptable for RMSEA = .17. Overall fit was deemed acceptable for interpretation. The scalar model did not significantly differ from the metric model (χ^2^ = 12.32 df = 12, *p* = .42) supporting the assumption of measurement invariance.

The first order CFA scalar model for the coping strategies scale was an acceptable fit to the data on (χ^2^ = 18.18 df = 8, χ^2^:df = 2.27, CFI =.99; TLI = .97; SRMR=.02) with the RMSEA=.14 above the threshold of acceptable. The scalar model did not significantly differ from the metric model (χ^2^ = 15.99 df = 9, *p* = .07) supporting the assumption of measurement invariance.

Measurement invariance was not run on memory characteristics, as it no longer had a latent factor structure.

# References

Chen, F. F. (2007). Sensitivity of goodness of fit indexes to lack of measurement invariance. *Structural Equation Modeling, 14*(3), 464-504.

Hu, L., & Bentler, P. M. (1999). Cutoff criteria for fit indexes in covariance structure analysis: Conventional criteria versus new alternatives. *Structural Equation Modeling: A Multidisciplinary Journal, 6*(1), 1-55. doi:10.1080/10705519909540118

Muthén, B. O., Muthén, L. K., & Asparouhov, T. (2017). *Regression and mediation analysis using Mplus*: Muthén & Muthén Los Angeles, CA.

Muthén, L. K., & Muthén, B. O. (2007). Mplus. *Statistical analysis with latent variables. Version, 3*.

Smith, K. V., & Ehlers, A. (2019). The Oxford Grief Study Cognitive Measures Explotatory and Confirmatory Factor Analyses and Psychometric Validation. *Manuscript in preparation.*

Van de Schoot, R., Lugtig, P., & Hox, J. (2012). A checklist for testing measurement invariance. *European Journal of Developmental Psychology, 9*(4), 486-492.

Wickrama, K. K., Lee, T. K., O’Neal, C. W., & Lorenz, F. O. (2016). *Higher-order growth curves and mixture modeling with Mplus: A practical guide*. New York, USA: Routledge.
